# In ovo injection of bee pollen extract on hatchability, chick quality, glycogen reserves and production performance in broiler chickens

**DOI:** 10.1016/j.psj.2024.104035

**Published:** 2024-06-26

**Authors:** Joanna Pawłowska, Ewa Sosnówka-Czajka, Joanna Nowak, Iwona Skomorucha, Katarzyna Połtowicz

**Affiliations:** Department of Poultry Breeding, National Research Institute of Animal Production, Balice 32-083, Poland

**Keywords:** in-ovo feeding, bee pollen, glycogen, chick quality, performance

## Abstract

This study aimed to investigate the effects of in ovo injection of bee pollen (**BP**) extract on some hatching traits, glycogen reserves and production performance in broilers. A total of 886 eggs was randomly assigned to 5 treatments: the 0.9% NaCl diluent-injected control group, and the groups that were injected with BP extract at 3 different concentrations: BP-1.5%, BP-2.5% and BP-5.0% (7, 12 and 25 mg/egg, respectively). The last group received a carbohydrate solution (**CS**). At 18 d of incubation, 500 µL of each solution was injected into the air sac of each egg of the injected groups. After hatching, a total of 570 one-day-old chicks were distributed into 5 groups (in ovo injection) with 3 replicates with 38 birds. The in ovo injection of BP at a dose of 5% and CS resulted in lower hatching rates (*P* < 0.05) as compared with the control group. The level of glycogen in the muscle tissue of newly hatched birds was not significantly different (*P* > 0.05). Supplementation of embryos with BP extract also caused a significant increase in the length of chicks in the BP-1.5% group (*P* < 0.001). Hatched chicks from the injected eggs had a lower liver glycogen content than that of the control. Based on the results, it was concluded that high-quality day-old chicks could be obtained by in ovo injection. Supplementation of chicks with 1.5% BP extract had a beneficial effect on BW during the first rearing period and showed a hypocholesterolemic effect in young chicks. At the same time, an adverse effect of a high dose of BP (5%) and a carbohydrate solution administered in ovo on the hatchability level of chicks was shown. The implementation of the carbohydrate mixture resulted in a deterioration of biochemical indices in the plasma of newly hatched chicks, especially in the case of transaminase activity.

## INTRODUCTION

Poultry production is one of the most important food sectors in agriculture. It is distinguished by the dynamic implementation of modern technological solutions. The genetic selection of poultry is also aimed at maximizing production, which is why modern lines of broiler chickens are selected towards a high growth rate and percentage of breast muscle in the carcass. As a result of selection, breast muscle yield has increased by 85% and 79%, respectively, in female and male broilers (Carney et al., 2022). Consequently, this results in increased metabolic needs of birds already during embryonic development (Buzała et al., 2015). In chicken embryos, glycogen reserves are depleted during incubation, leading to a temporary inhibition and restriction of chicken embryonic development (Givisiez et al., 2020). As part of poultry practice, compensation for these losses occurs once the hatchlings are transported to the farm, which can take up to 72 h after hatching. Furthermore, day-old chicks are the end product of the hatchery and are the starting material for commodity production. With economic aspects in mind, the main objective of commercial hatcheries is not only to obtain a high hatchability rate, but also healthy and fully developed chicks. Considering the mass production of broiler chickens and their short rearing time, even a slight improvement in energy status during the peri-hatching period can have a positive impact on the condition and quality of the chicks and on the broiler production performance.

The introduction of bioactive substances into the environment of the developing chicken embryo (in ovo technology) as supplementation of broiler chickens during embryogenesis is becoming increasingly important in poultry production (Saeed et al., 2019). Phytobiotics, and in particular bee pollen, due to their natural origin, multidirectional effects and no withdrawal period, appear to be particularly useful as a tool in improving chicks quality and broiler chicken productivity. However, to demonstrate the long-term impacts of in ovo supplementation and its financial advantages for the poultry industry, further research is necessary.

Bee products are considered to be natural and safe additives, and their multidirectional action offers opportunities for widespread use in animal production. The results of a number of studies indicate a beneficial effect of bee pollen on animal productivity (Abdelnour et al., 2019). It is also believed that feed additives in the form of bee products can improve feed palatability and thus increase feed intake and improve production performance, including feed conversion and weight gain in poultry (Haščík et al., 2012; Attia et al., 2014; Fazayeli-Rad et al., 2015; Farag and El-Rayes, 2016; Hosseini et al., 2016; Saeed et al., 2018).

To date, consideration of bee pollen application in poultry production has focused on its use as a feed additive in bird nutrition. However, the prices of natural additives, especially those of bee origin, are high due to their small quantities, as well as the methods of their extraction. In addition, their sterilization and methods of transformation into final products, such as extracts, require a highly sophisticated technology, which also contributes to higher production costs. Given these factors, an alternative method of supplementing broilers may be to use in ovo technology involving the delivery of small amounts of bioactive substances directly to the developing embryo. Despite the extensive literature on the inclusion of bee products in the diet of chickens, there is little information on the use of the in ovo method for the administration of bee pollen. Based on the current study, the research hypothesis was that the in ovo administration of bee pollen extract, due to the biologically active compounds it contains, would have a beneficial effect on hatching parameters, muscle and liver glycogen content and biochemical parameters, as well as production performance and slaughter yield of broiler chickens. For the purpose of verifying the above hypothesis, an experiment was conducted with the aim of in ovo injection of bee pollen extract at 3 different doses. For comparison purposes, a carbohydrate solution with an analogous content of simple and complex sugars was administered with the same method.

## MATERIALS AND METHODS

### Ethics Statement

The protocol for animal experimental procedures was approved by the Local Ethics Committee for Animal Experimentation in Kraków (158/2018, 16 July 2018).

### Experimental Animals and Management

The experiment used 886 hatching eggs from broiler breeder strain (Ross 308) at 38 wk of age. Eggs were individually weighed, and egg weight averaged 61.6 g. Eggs were incubated in an incubator (HEKA Brutgeräte, Rietberg, Germany) according to standard hatchery practices with a temperature (**T**) of 37.8°C and relative humidity (**RH**) of 55% from d 1 to 18. On the 18th d of incubation, the eggs were transferred from the setter tray to the hatcher basket, with an incubation T of 37.2°C and RH of 65%. After hatching, the chicks were placed in pens on litter and fed ad libitum the same complete unrestricted feed mixes of starter - up to wk 3 (EM 3,000 kcal, CP 21%), grower - from wk 4 to wk 5 (EM 3,100 kcal, CP 19.8%) and finisher at wk 6 (EM 3,100 kcal, CP 18.5%; Table 1) (Smulikowska and Rutkowski, 2018).

**Table 1:** Composition of feed mixtures used in the study.

Ingredients (%)	Diet	
	Starter^1^	Grower^2^ Finisher^3^
Corn grain	55.35	47.21
Soybean meal	37.50	33.75
Wheat	-	10.00
Canola oil	2.90	4.80
Fodder chalk	1.10	1.15
Calcium (II) phosphate	2.10	2.00
Sodium chloride	0.30	0.30
L-Lysine	0.03	0.09
DL-Methionine	0.22	0.20
Vitamin-mineral premix	0.50	0.50

1 Supplements per kg starter diet: vit. A 13,500 IU (retinol); vit. D3 3,600 IU; vit. E 45 IU (dl-alpha-tocopherol); vit. B1 3.25 mg; vit. B2 7.5 mg; vit. B6 5 mg; vit. B12 32.5 mcg; vit. K3 3 mg; biotin 150 mcg; niacin 45 mg, pantothenic acid 15 mg, folic acid 1.5 mg; choline chloride 447.6 mg; Mn 100 mg; Cu 1.75 mg; Fe 76.5 mg; Se 0.75 mg; I 1 mg; Zn 75 mg; Co 4 mg; Seldox HM (antioxidant) 25 mg; Clinacox 0.5% 1 mg.

2 Supplements per kg grower diet: vit. A 12,000 IU (retinol); vit. D3 3,250 IU; vit. E 44 IU (dl-alpha-tocopherol); vit. B1 2 mg; vit. B2 7.3 mg; vit. B6 4.25 mg; vit. B12 30 mcg; vit. K3 2.25 mg; biotin 100 mcg; niacin 40 mg; pantothenic acid 12 mg; folic acid 1.0 mg; choline chloride 335.7 mg; Mn 100 mg; Cu 1.5 mg; Fe 65 mg; Se 0.25 mg; I 0.9 mg; Zn 65 mg; Co 0.4 mg; Seldox HM (antioxidant) 25 mg; Coxidin 200 100 mg.

3 Supplements per kg finisher diet: vit. A 10,500 IU (retinol); vit. D3 2,500 IU; vit. E 44 IU (dl-alpha-tocopherol); vit. B1 1.5 mg; vit. B2 5.5 mg; vit. B6 3.25 mg; vit. B12 25 mcg; vit. K3 2 mg; biotin 100 mcg; niacin 35 mg; pantothenic acid 12 mg; folic acid 1 mg; choline chloride 298.4 mg; Mn 80 mg; Cu 8 mg; Fe 60 mg; Se 0.2 mg; I 0.8 mg; Zn 50 mg; Co 0.4 mg; Seldox HM (antioxidant) 25 mg.

### Preparation of Treatment Solutions

Entomophilus Pollen Dry Extract Entolase, No. SPOL001 (B Natural, Corbetta MI, Italy), was used in the study. The in ovo injection procedure was preceded by analysis of the chemical composition of the bee pollen (**BP**) extract at Eurofins Food Testing Laboratory. The following were determined in the BP: pH (PN ISO 2917:2001, potentiometrically), sugar profile (high-performance liquid chromatography with refractometric detection; HPLC-RID, vitamin E (EN 12822: 2000, LC-FLD), vitamin A (EN 12823-1 2014, LC-DAD), beta-carotene (EN 12823-2:2000, LC-DAD), vitamin C (food chemistry, 94626-631, LC-DAD), polyphenols (in-house method, spectrophotometrically as gallic acid equivalent). The remaining chemical composition was provided by the BP producer (Table 2). For in ovo injection, 1.5, 2.5, and 5% aqueous extracts of BP were prepared. Based on the results of quantitative analysis of simple and complex sugars in BP, a solution of analogous carbohydrate composition containing fructose (9.6%), glucose (9.5%) and maltose (6.9%) was also prepared. The prepared solutions were stirred using a magnetic stirrer type ES21H (WIGO, Pruszkow, Poland) at 1,000 rpm at room temperature for 30 min. The volume of the solution was 500 µL/egg, and the diluent was 0.9% NaCl solution (Gilbert, France). All experimental solutions were prepared on the day of injection. Before the in ovo injection procedure, all solutions were warmed to 37°C.

**Table 2:** Composition of bee pollen extract.

Item	
Protein (%)	10
Fat (%)	2.5–5.0
Crude fiber (%)	2.7
Ash (%)	< 10
Carbohydrates (%)	65
Total sugar (%):	26
glucose	9.5
fructose	9.6
sucrose	< 0.05
maltose	6.9
Ph	4.8
Energy value (kcal)	330
Energy value (KJ)	1380
Vitamin E (IU/kg)	46.34
Vitamin A (IU/kg)	< 700 LOQ^1^
Vitamin C (mg/100g)	< 0.5 LOQ^1^
Beta-carotene (µg/100g)	< 0.5 LOQ^1^
Polyphenols (mg/kg)	10500
Heavy metals (ppm):	
Cadm	< 1
Arsenic	< 3
Mercury	< 0.1
Lead	< 3
Microbiological analysis:	
Aerobic plate count (CFU^2^/g)	< 5*10000
Total yeast and mold counts (CFU^2^/g)	< 5*100
Gram negative bacteria (CFU^2^/g)	< 100
*Escherichia coli*	not present
*Staphylococcus aureus*	not present
*Salmonella*	not present
Aflatoxins B1 (ppb)	< 10
Aflatoxins B1, B2, G1, G2 (ppb)	< 5

1 LOQ, limit of quantitation.

2 CFU, colony forming units.

### In Ovo Injection

On the 18th day of incubation, the eggs were candled to eliminate unfertilized eggs and those containing dead embryos. The eggs containing live embryos were randomly assigned to one of five treatment groups (165 eggs per group), with eleven replicates per treatment and 15 eggs per replicate: the control group consisted of eggs that were injected with 500 µl of saline (0.9%) and, groups that were injected with 500 µL of BP extract at 3 different concentrations: BP-1.5%, BP-2.5% and BP-5.0% (7, 12 and 25 mg/egg, respectively). The last group received a carbohydrate solution (**CS**). The manipulation was carried out into the air cell of the egg through a hole in the shell using a 21-gauge needle, after disinfection with a 70% ethanol solution. After the injection, the hole was sealed with Parafilm® tape, and the eggs were transferred to hatching baskets for continued incubation (Lis et al., 2009).

### Quality of One-Day Old Chicks

On the day of hatching, the live chicks were weighed individually on an electronic scale to the nearest 0.01 g and the average BW was calculated, and the hatchability (%) was determined from the eggs laid for each group. To minimize the influence of human factor, all hatched day-old chicks were subjected to quality assessment by 2 evaluators. The following parameters were used: hatching weight, Tona score and chick length. The scoring evaluation was performed according to the methodology developed by Tona et al. (2003), with evaluation of activity, external appearance and membrane residue. These traits were evaluated according to their importance on a 100-point scale. Chick length was determined by measuring the length from the tip of the beak to the end of the middle toe of the leg to the nearest 0.5 cm (Ketels, 2011).

### Tissue Sampling and Glycogen Analysis

Determination of liver and muscle glycogen content was carried out at the Central Laboratory of the National Research Institute of Animal Production (Balice, Poland). On the 1st and 7th day of life, 15 birds per treatment (5 chicks from every replicate) were euthanized to measure glycogen content of the right thigh muscle and liver. Immediately after collection, the tissues were frozen in liquid nitrogen. The collected liver and muscle tissues were subjected to protein hydrolysis, glycogen precipitation and cleaning according to the methodology (Rasouli et al., 2015). Glycogen content was determined using the Anthrone method based on the methodology of Chun & Yin (1998).

### Blood Samples and Biochemical Analysis

From each experimental group, blood samples were taken from 12 randomly selected birds (4 chicks from every replicate) on the 1st, 7th and 42nd day of life for the determination of selected blood biochemical parameters. Determinations of blood biochemical indices were performed with a Mindray BS-120 biochemical analyzer using reagent kits and methodology from Alpha Diagnostics Inc. (Warsaw, Poland): total protein (**TP**) by colorimetric, biuret method; glucose (**GLU**) by colorimetric, enzymatic method with glucose oxidase (**GOD/PAP**); total cholesterol (**TC**) by colorimetric, enzymatic method with esterase and cholesterol oxidase (**CHOD/PAP**); high density lipoprotein-cholesterol (**HDL-c**) and low density lipoprotein-cholesterol (**LDL-c**) fraction by direct colorimetric method by selective blocking; uric acid (**UA**) by colorimetric, enzymatic method with uricase; aspartate transaminase (**AST**) and alanine transaminase (**ALT**) by UV kinetic method according to IFCC recommendations, without activation with pyridoxal phosphate.

### Growth Performance

A total of 549 1-day-old chicks from the incubation experiment were distributed into 5 treatments (in ovo injection) with 3 replicates (from 32 to 38 eggs per replicate). During the experiment, performance parameters including daily feed intake (**FI**) and weekly BW were recorded. The average BW at 7, 21 and 42 d of life and FI, body weight gain (**BWG**), feed conversion ratio (**FCR**; kg/kg weight gain), and mortality (%) for the entire experimental period (1 to 42 d) were calculated for each group.

### Determination of Carcass Characteristics

On the 42nd day of life, 30 birds (15 pullets and 15 cockerels) with the BW close to the average weight for the group were selected for slaughter. The animals were deprived of access to feed for 12 h, slaughtered, then defeathered and eviscerated. The obtained carcasses were weighed before as well as after 24-h refrigeration at 4°C and the weight of the carcass without giblets was determined. The cooled carcasses were subjected to a simplified slaughter analysis. During dissection, the following carcass elements were extracted: pectoral muscles (total of 2 superficial and deep muscles), muscles of both legs (total of thigh and drumstick muscles), abdominal fat, and edible giblets (liver, gizzard and heart). The obtained carcass parts were weighed on an electronic scale with an accuracy of 0.1 g. Based on the results of the slaughter analysis, the percentage slaughter yield without giblets was calculated as the ratio of the weight of the eviscerated, chilled carcass without giblets to the live BW. On the basis of the slaughter analysis, the proportion of breast and leg muscle, leg bone, giblets and abdominal fat in the carcass with giblets were calculated.

### Statistical Analysis

The procedures were performed using the Statistica software version 13.3 (StatSoft Inc., Tulsa). The data were analyzed using one-way ANOVA with Duncan's multiple range test, or when the variables did not meet the assumptions of a normal distribution the Kruskal-Wallis test with the correction of multiple comparisons of mean ranks for all samples. In order to determine the influence of the 2 factors (in ovo injection and age) on day 1st and 7th, the *p*-values were determined using 2-way ANOVA. Differences were considered statistically significant at a significance level of *P* < 0.05.

## RESULTS

### Incubation and Newly Hatched Chick Quality

In ovo supplementation with BP extract at a dose of 1.5% and 2.5% had no significant effect on the hatchability percentage compared to the control group, although a tendency to increase this parameter in the BP-1.5% group (hatchability above 90%) can be noted (Table 3). The lowest number of chicks hatched in the group injected with BP was at a dose of 5% (*P* < 0.05). There was a higher BW (3% on average) in the BP-1.5% and CS groups compared to the control group (*P* < 0.05). Supplementation of embryos with BP extract also caused a significant increase in the length of chicks in the BP-1.5% group compared to the control group (*P* < 0.001). Chicks from the aforementioned group were longer by 0.4 cm on average. On the other hand, analyzing the Tona score, a tendency toward lower scores was observed in the group supplemented with CS, in which the average number of points awarded was 93.6, and was 4.8 points (or 5%) lower compared to the control group (*P* > 0.05).

**Table 3:** Effect of in ovo injection of bee pollen extract or carbohydrates solution on hatchability, BW and quality of day-old chicks.

Group^1^	Variables			
	Hatchability (%)	BW (g)	Tona score	Chick length (cm)
Control	83.3^a^	45.96^b^	98.38	14.33^b^
BP-1.5%	91.7^a^	46.26^a^	97.19	14.72^a^
BP-2.5%	83.3^a^	45.56^b^	95.78	14.62^ab^
BP-5.0%	58.3^b^	45.05^b^	95.86	14.43^ab^
CS	67.1^b^	47.47^a^	93.63	14.53^ab^
SEM	4.02	0.17	0.42	0.05
*P*-value	0.012	0.011	0.405	< 0.001

1 Control, in ovo injection of 0.9% of saline; BP-1.5%, in ovo injection of 1.5% bee pollen extract; BP-2.5%, in ovo injection of 2.5% bee pollen extract; BP-5.0%, in ovo injection of 5% bee pollen extract; CS, in ovo injection of carbohydrates solution.

a-b Means within a column lacking a common superscript differ (*P* < 0.05).

### Glycogen Reserves

As expected, glycogen content in muscle tissue and the liver (Table 4) was significantly higher in older broilers (*P* < 0.05). The in ovo × day interactions for glycogen in the muscle tissue were not significant. Irrespective of day, in ovo injection caused a decrease in glycogen levels when CS or BP-1.5% was administered. Reserves of glycogen in the CS group were more than 2 times lower compared to the control group (*P* < 0.05). A 2-way ANOVA test revealed a significant interaction between the effect of in ovo injection and age in the liver. The interaction was mainly due higher glycogen content on d 7 of rearing. Moreover, the highest glycogen levels found in supplemented group (BP-2.5% and BP-5.0%) on d 7 of rearing compared to both non-supplemented groups. A similar relationship was noted irrespective age, where chickens from the BP-2.5% and BP-5.0 had significantly higher levels of glycogen in the livers in comparison with birds from the control group (*P* < 0.05).

**Table 4:** Effects of age and in ovo injection of bee pollen extract or carbohydrates solution on glycogen reserves of chickens during the early rearing period.

		Variables^2^	
Factors		Muscle	Liver
Age			
1		54.59^b^	109.41^b^
7		141.97^a^	693.80^a^
In ovo^1^			
Control		138.61^ac^	452.60^b^
BP-1.5%		75.45^b^	312.76^bc^
BP-2.5%		183.80^a^	879.16^a^
BP-5.0%		108.97^bc^	773.93^a^
CS		57.06^b^	75.92^c^
Age x In ovo			
Age 1	Control	67.40	224.02^bc^
	BP-1.5%	57.81	117.03^bc^
	BP-2.5%	66.34	43.26^c^
	BP-5.0%	58.23	118.35^bc^
	CS	23.17	44.42^c^
Age 7	Control	174.21	566.89^b^
	BP-1.5%	84.26	410.63^bc^
	BP-2.5%	242.52	1297.12^a^
	BP-5.0%	134.34	1101.72^a^
	CS	74.01	91.67^bc^
SEM		10.61	68.62
*p*-value			
Age effect		< 0.001	<0.001
In ovo effect		0.001	0.001
Interaction		0.056	< 0.001

1 Control, in ovo injection of 0.9% of saline; BP-1.5%, in ovo injection of 1.5% bee pollen extract; BP-2.5%, in ovo injection of 2.5% bee pollen extract; BP-5.0%, in ovo injection of 5% bee pollen extract; CS, in ovo injection of carbohydrates solution.

a-c Means within each column lacking a common superscript differ (*P* < 0.05).

### Blood Biochemical Analysis

The average level of selected biochemical indices in the plasma of broilers is shown in Table 5. A significant interaction between in ovo injection and age was observed for TP, TC, HDL-c, LDL-c, UA, ALT, and AST. In the case of TP, experimental administration of 5% BP extract and CS in ovo increased the value of TP in the serum of day-old chicks compared to the control group (P < 0.05). At d 7, chicks injected with BP extract had reduced blood TP concentrations compared to the control and CS chicks by an average of 24%. For TC, a significantly higher levels were observed in day-old chicks. As for the blood lipid profile, there was a significant in the HDL-c fraction in newly hatched chicks. For this parameter, a significant in ovo × day interactions was also observed, with increase the CS group and a decrease in the BP-1.5% and BP-2.5% groups in the plasma of one day old chicks by an average of 0.9 mmol/l with respect to the non-supplemented group. Irrespective of age, the concentration of total TC and LDL-c fraction did not differ between the groups (*P* > 0.05). In addition to the TP and lipid profile, the analysis of GLU in the plasma of chicks immediately after hatching was also performed. As shown, the concentration of this parameter was similar in all experimental groups (*P* > 0.05). The difference in GLU levels resulted only from the day of rearing. In contrast, UA levels was influenced by the interaction of in ovo and age (*P* < 0.05). Birds in the CS group showed lower UA content (P < 0.05) compared to the control group. In chickens on d 7 of age, the in ovo administration of BP extract caused a marked decrease in blood UA concentrations in the BP-1.5%, BP-2.5% and BP-5.0% groups by 23%, 33% and 44%, respectively (P < 0.05). Comparing the results of biochemical analyses in the serum of broiler chickens, it was found that supplementation of embryos with CS increased ALT activities and the measurement increased with age (*P* < 0.05). In contrast, injection of BP had no effect on the activity of transaminases (*P* > 0.05).

**Table 5:** Effects of age and in ovo injection of bee pollen extract or carbohydrates solution on blood serum parameters of chickens during the early rearing period.

		Variables^2^							
Factors		TP (g/dL)	TC (mmol/l)	HDL-c (mmol/l)	LDL-c (mmol/l)	GLU (mmol/l)	UA (µmol/l)	ALT (IU/l)	AST (IU/l)
Age									
1		2.17^b^	10.24^a^	4.14^a^	3.80^a^	11.84^b^	245.44^b^	9.44	129.7^b^
7		3.03^a^	4.64^b^	3.23^b^	1.34^b^	14.30^a^	484.18^a^	9.80	216.3^a^
*In ovo*^1^									
Control		2.82^a^	8.14	4.11^ab^	2.96	13.39	463.04^a^	8.40	163.3^b^
BP-1.5%		2.19^b^	7.27	3.18^c^	2.42	12.25	350.17^ab^	10.00	149.5^b^
BP-2.5%		2.20^b^	7.23	3.12^c^	2.50	14.34	325.47^cb^	9.95	157.3^b^
BP-5.0%		2.80^a^	7.10	3.81^b^	2.31	13.29	437.71^a^	9.04	172.2^b^
CS		3.15^a^	7.47	4.43^a^	2.69	11.63	197.46^e^	11.28	238.1^a^
Age x In ovo									
Age 1	Control	2.06^c^	10.29^a^	4.25^b^	3.78^a^	12.49	200.29^c^	6.87^b^	106.7^b^
	BP-1.5%	1.64^c^	10.25^a^	3.35^cd^	3.74^a^	11.06	141.21^c^	8.59^b^	93.9^b^
	BP-2.5%	1.70^c^	10.02^a^	3.42^cd^	3.83^a^	11.59	165.11^c^	9.83^b^	102.5^b^
	BP-5.0%	2.90^b^	9.40^a^	4.33^b^	3.37^a^	12.66	470.50^bd^	8.12^b^	125.6^b^
	CS	2.70^b^	11.63^a^	5.88^a^	4.49^a^	11.18	252.11^c^	15.54^a^	240.6^a^
Age 7	Control	3.59^a^	5.99^b^	3.97bde	2.15^b^	14.28	725.80^a^	10.10^b^	226.2^a^
	BP-1.5%	2.75^b^	4.29^bc^	3.00^c^	1.10^c^	13.45	559.13^b^	11.25^b^	198.8^a^
	BP-2.5%	2.70^b^	4.44^bc^	2.83^c^	1.17^c^	17.08	485.83^bd^	10.06^b^	205.9^a^
	BP-5.0%	2.70^b^	4.79^bc^	3.28^ce^	1.26^c^	13.93	404.92^d^	9.96^b^	218.7^a^
	CS	3.61^a^	3.30^c^	2.98^c^	0.89^c^	12.07	142.81^c^	7.02^b^	235.7^a^
SEM		0.08	0.34	0.11	0.16	0.34	24.23	0.50	7.56
*p*-value									
Age effect		< 0.001	< 0.001	< 0.001	<0.001	< 0.001	< 0.001	0.905	< 0.001
In ovo effect		< 0.001	0.333	< 0.001	0.130	0.063	< 0.001	0.429	< 0.001
Interaction		< 0.001	0.012	< 0.001	0.017	0.116	< 0.001	0.002	0.002

1 Control, *in ovo* injection of 0.9% of saline; BP-1.5%, *in ovo* injection of 1.5% bee pollen extract; BP-2.5%, *in ovo* injection of 2.5% bee pollen extract; BP-5.0%, *in ovo* injection of 5% bee pollen extract; CS, *in ovo* injection of carbohydrates solution.

2 TP, total protein; TC, total cholesterol; HDL-c, high density lipoprotein-cholesterol; LDL-c, low density lipoprotein cholesterol; GLU, glucose; UA, uric acid; ALT, alanine transaminase; AST, aspartate transaminase.

a-e Means within each columns lacking a common superscript differ (*P* < 0.05).

### Broiler Chicken Performance

The effects of in ovo injection on BW of chickens at different ages are presented in Table 6. On the 7th day of life, analogous to the first day after hatching, the highest average BW was observed in chickens of the BP-1.5% group compared to the control group (*P* < 0.05). On the 21st day of life of the birds, despite the lack of statistical significance, an increased BW of 7% was also observed in the BP-1.5% group compared to the control group. In contrast, supplementation with BP or CS at the embryogenesis stage showed no significant effect on the BW of the birds at d 35 with respect to the control group. However, on d 42 of rearing, statistically significant intergroup variation in mean BW was noted. The highest BW at the end of rearing was observed in chicks from the CS group, and the lowest in the BP-2.5% group. The difference between the groups averaged 466 g at *P* < 0.05.

**Table 6:** Effect of in ovo injection of bee pollen extract or carbohydrates solution on BW (g) of chickens at different ages.

Group^1^	Day			
	7	21	35	42
Control	127.0^b^	789.1	2305.6	3103.1^b^
BP-1.5%	133.1^a^	848.6	2324.3	3055.2^bc^
BP-2.5%	131.4^ab^	774.3	2235.4	3027.8^c^
BP-5.0%	123.8^bc^	741.1	2312.4	3128.3^b^
CS	126.2^b^	728.8	2403.0	3493.9^a^
SEM	0.89	36.83	14.60	18.27
*P*-value	< 0.001	0.078	0.478	< 0.001

1 Control, in ovo injection of 0.9% of saline; BP-1.5%, in ovo injection of 1.5% bee pollen extract; BP-2.5%, in ovo injection of 2.5% bee pollen extract; BP-5.0%, in ovo injection of 5% bee pollen extract; CS, in ovo injection of carbohydrates solution.

a-c Means within a column lacking a common superscript differ (*P* < 0.05).

Table 7 shows the effects of in ovo injection of BP or CS on performance of broiler chickens (0–6 wk). Based on the analysis of the data for the entire rearing period, a clear advantage of production parameters was observed in chickens given the CS in ovo method. Statistically significant differences between them and the chickens of the other groups concerned weight gain and FCR.

**Table 7:** Effect of *in ovo* injection of bee pollen extract or carbohydrates solution on production results of chickens (1–42 d of age).

	Variables^2^			
Group^1^	BWG (g)	FI (g)	FCR (kg/kg weight gain)	Mortality (%)
Control	3057.1^b^	4402.0	1.43^a^	5.1
BP-1.5%	3008.9^b^	5880.5	1.95^a^	5.0
BP-2.5%	2982.2^b^	4369.4	1.46^a^	9.8
BP-5.0%	3083.2^b^	6066.2	1.97^a^	12.8
CS	3446.4^a^	4439.8	1.30^b^	3.3
SEM	54.02	382.10	0.03	0.08
*P*-value	0.034	0.499	0.090	0.091

1 Control, in ovo injection of 0.9% of saline; BP-1.5%, in ovo injection of 1.5% bee pollen extract; BP-2.5%, in ovo injection of 2.5% bee pollen extract; BP-5.0%, in ovo injection of 5% bee pollen extract; CS, in ovo injection of carbohydrates solution.

2 BWG, body weight gain; FI, feed intake; FCR, feed conversion ratio.

a-b Means within a column lacking a common superscript differ (*P* < 0.05).

### Carcass Analysis

The effects of in ovo supplementation with BP or CS on traits determining slaughter performance and the proportion of individual elements in broiler carcasses is shown in Table 8. Analyzing the results of the slaughter analysis, there was a difference in carcass yield between the BP-2.5% and control groups at *P* < 0.05. A significantly lower proportion of giblets in the carcass was observed in all injected groups (*P* < 0.05). There was no effects of the in ovo injection on breast and leg yield, and abdominal fat (*P* > 0.05).

**Table 8:** Effects of in ovo injection of bee pollen extract or carbohydrates solution on carcass characteristics of broilers at 42 d of age.

Group^1^	Variables (%)				
	Carcass yield	Breast yield	Leg yield	Giblets	Abdominal fat
Control	74.66^b^	31.99	21.02	3.80^a^	1.41
BP-1.5%	75.19^ab^	32.58	21.32	3.67^b^	1.44
BP-2.5%	75.96^a^	31.94	22.00	3.63^b^	1.32
BP-5.0%	75.79^a^	32.08	27.42	3.59^b^	1.52
CS	75.41^ab^	32.23	22.10	3.46^b^	1.34
SEM	0.15	0.16	1.42	0.03	0.03
P-value	0.042	0.489	0.381	< 0.01	0.504

1 Control, in ovo injection of 0.9% of saline; BP-1.5%, in ovo injection of 1.5% bee pollen extract; BP-2.5%, in ovo injection of 2.5% bee pollen extract; BP-5.0%, in ovo injection of 5% bee pollen extract; CS, in ovo injection of carbohydrates solution.

a-b Means within a column lacking a common superscript differ (*P* < 0.05).

## DISCUSSION

Hatchability is one of the most important indicators to assess the effectiveness of in ovo injection (Slawinska et al., 2020). In our study, the administration of high doses of BP (5%) in the form of extract or CS into the air cell of the developing chicken embryo resulted in a significant reduction in hatchability levels. The results obtained confirm the results of Zhai et al. (2011b), who injected carbohydrate solution by the in ovo method at 18.5 d of incubation and observed that increasing the volume of the solution negatively affected the hatchability of broiler chicks. The same opinion was held by Tasharofi et al. (2018) who claimed that by injecting high doses of carbohydrates, the energy metabolism of the embryo may be overloaded and consequently the number of chicks hatched may decrease. The mechanism for the significant deterioration in hatchability observed in our study, following the introduction of a high dose of pollen and CS, may have been related to the high percentage concentration of fructose. Research showed that the use of high doses of fructose injection in ovo significantly worsened the hatchability of chicks as well as their BW (Zhai et al., 2011a; Kucharska-Gaca et al., 2017). It is worth noting that in the experiment in question, the content of the aforementioned simple sugar in the BP extract was 9.6%. It can also be speculated that the higher mortality in the aforementioned groups may have been due to the high osmolality of the solution, which was not determined in the present study.

It is also worth noting that a general deterioration in body function was observed in the BP-5.0% and CS groups, which was expressed in higher values of blood biochemical parameters in day-old chicks. In the aforementioned groups, high concentrations of plasma proteins, which are important in maintaining the body's homeostasis, were recorded, with reference values for this parameter at 1.4 to 3.8 g/dL for hens from 1 to 4 wk of age (Mazurkiewicz and Wieliczko, 2019). This is confirmed by the study of Taha et al. (2019), who reported an increase in protein in the blood plasma of chickens given 0.5 mL royal jelly on d 7 of incubation. Farag & El-Rayes (2016) observed that the introduction of 0.6% BP into the chicken mix resulted in an increase in blood TP levels. In the current study the correlation between the concentration of BP extract and the level of HDL fraction in the blood plasma of newly hatched chicks was also observed. The highest level of the aforementioned lipoprotein was recorded in birds given BP extract at a dose of 5 g/100 mL and injected with a CS. In contrast, the lowest HDL fraction was observed in groups with lower pollen concentrations (1.5% and 2.5%). The above results correspond with those of a study in which geese that were fed with feed supplemented with 10% fructose had higher levels of triglycerides and lipoprotein subfractions in the blood relative to the control birds (Wei et al., 2022).

Comparing low effectiveness of BP on blood indices and glycogen reserves on d 1 of life with the results obtained on d 7 of rearing, it can be assumed that the positive effects of hen embryo supplementation are only apparent after the first week of birds' rearing. This may indicate that the chicks only start to use the bioactive substances contained in the BP more intensively after hatching. Before hatching, the chicken embryo uses the yolk sac, which is rich in fats, while after hatching it starts to take in solid feed rich in protein and carbohydrates. Importantly, during these first days of life, the remains of the yolk sac are metabolized. It has been observed that chicks gradually gain the ability to absorb more simple sugars and amino acids after hatching (Noy and Sklan, 1999; Jha et al., 2019). According to the literature, on the day of hatching, the chick has a GLU and amino acid absorption capacity of 43 to 53%, while this level increases as the young bird grows to reach 80% by the fourth day (Noy and Sklan, 1999). Nitsan et al. (1991) demonstrated that the specific activity of pancreatic enzymes in slaughter chickens increases gradually to reach a peak at d 11 of rearing. Based on the results of a study conducted on broiler chickens given 3 mg/egg ascorbic acid on d 17 of incubation, increased daily weight gains were observed during the third and fourth week of rearing (Zhang et al., 2019).

Phytogenic feed additives can reduce serum cholesterol levels in animals (Karásková et al., 2015). According to Bölükbaşi et al. (2008), the reduction in cholesterol concentration is related, among other things, to the inhibition of the activity of the liver enzyme HMG-CoA reductase by the essential oil components found in plants. The apparent effect of a BP supplemented diet on lowering lipids and raising HDL was reported, among others, by Farag et al. (2014). The hypocholesterolemic effect of BP extract was also evident in the present in-house experiment on d 7 of rearing, as expressed by low HDL, LDL and TC concentrations in the injected birds. Similar results to the present experiment were obtained by Taha et al. (2019), who observed that the administration of 0.25- or 0.5-mL royal jelly/egg on d 7 of incubation resulted in a significant reduction in the serum levels of TC, HDL and LDL in the birds. The results of some studies suggest that the mechanism for this effect of BP may be due to its high polyphenol content. Indeed, Braakhuis (2019) found that polyphenols inhibit numerous cellular enzymes, such as lipoxygenase, xanthine oxidase and phospholipase, and reduce lipoprotein peroxidation. It should be noted, however, that in our own experiment the hypocholesterolemic effect was also noted in the group in which carbohydrates were administered in ovo, which may indicate that it is the administration of these compounds, particularly GLU, which promotes the reduction of blood lipid parameters. Hussein et al. (2016) showed that the addition of sugar syrup (5%) to the diet of broiler chickens leads to a reduction in LDL and TC compared to birds fed a conventional diet. However, these conclusions require more detailed research work to clarify the mechanism and significance of these observations.

A significant effect of injecting 1.5% BP extract to increase the weight and body length of newly hatched chicks was found in the study. Similar results were obtained by Memon et al. (2019), who observed a trend towards increased BW in chicks supplemented with honey during the embryonic period or the first week of rearing. In the case of the experiment in question, the chicks given 1.5% BP extract were as much as 0.4 cm longer compared to the control group. This was, among other things, the result of the high carbohydrate content of BP, which is also confirmed by numerous scientific publications (Chen et al., 2009; Zhai et al., 2011a Zhai et al., 2011b; Salmanzadeh, 2012). An increased BW of chicks (by 3.2%) was also obtained after the use of a synthetic CS for in ovo injection. At the same time, despite the lack of statistical confirmation, the group receiving the CS had the lowest Tona score (93.6/100). It is interesting, however, that the use of the above solution only resulted in higher BW, while 1.5% BP extract had a positive effect not only on BW, but also on the length of the chicks. This may indicate the positive effect of BP extract through its rich nutritional composition.

BP is thought to facilitate digestion and improve absorption in broilers (Wang et al., 2007). Interestingly, the higher quality of the newly hatched BP-1.5% chicks did not correlate with muscle and liver glycogen content. In the present experiment, it was hypothesized that BP could be a potential source of substrates for gluconeogenesis in the liver. However, in the present study, the use of in ovo injection significantly reduced liver glycogen levels in day-old chicks, while Kornasio et al. (2011) observed that chicks that were supplemented in ovo with a mixture of dextrin and β-hydroxy-β-methylbutyrate had increased liver and muscle glycogen content. Similar results were obtained by Yu et al. (2018), who found that the administration of an amino acid (arginine) at 17.5 d of incubation had a positive effect on early energy metabolism. In addition, Shafey et al. (2010) confirmed that administration of carnitine at 25, 50, 100, 200, 300, 400 and 500 µg/egg results in a linear increase in energy reserves in the form of glycogen in the liver and muscle. In contrast, Uni et al. (2005) report that the injection of carbohydrates into the egg did not significantly affect the glycogen content of the birds' muscles.

According to Christensen et al. (2001), increased glycogen stores are associated with higher weights of day-old chicks, which was not observed in our experiment. The lack of an effect in terms of increased liver and muscle glycogen content is related to increased plasma TP levels in chicks from the BP-5.0% and CS groups and UA in the BP-5.0% group. According to Pulikanti et al. (2010), between d 15 and 19 of bird incubation, liver metabolism is activated to supply GLU and fatty acids to the embryonic muscles. Furthermore, during the peri-hatching phase, carbohydrates are metabolized rather than lipids. Consequently, this results in a high consumption of glycogen, which in turn triggers a degradation reaction of muscle proteins and amino acids for gluconeogenesis. It can therefore be assumed that due to the high availability of sugar from in ovo supplementation (BP-5.0% and CS group), the mechanism of embryonic gluconeogenesis was inhibited. This is confirmed by the study of Zamani et al. (2019), who, in contrast to changes in the liver, reported no significant difference in glycogen content in the leg and neck muscles of ostrich chicks given a carbohydrate solution (20% maltose, 2.5% sucrose and 2.5% dextrin) in ovo. According to Edwards et al. (1999), skeletal muscle sensitivity is low to active ingredient supplementation, despite differences in liver glycogen reserves.

As shown in the present experiment, the application of a 1.5% dose of BP had a positive effect on the BW of the birds on d 1 and 7 of life. These results are consistent with those reported in other studies using different bee products in ovo (Memon et al., 2019; Medeiros Dal'Alba et al., 2020). Similar observations were made by Gohari et al. (2010), who reported that royal jelly injection at 0.5 and 1 mg/egg significantly increased the BW of broilers in the third week of rearing. It is also noteworthy that in the groups receiving doses of 1.5% and 2.5% of BP in ovo, no deaths of chickens were recorded in the first rearing period. It can be assumed that the active substances contained in BP have a beneficial effect on bird health and immune response (Attia et al., 2017). However, the stimulating effect of BP injection on growth parameters of broilers on d 42 of rearing has not been reported. According to Zhan et al. (2007), any advantage in early growth is mostly lost as the birds get older, which may be partly due to the phenomenon of compensatory growth in broilers.

Administration of BP extract in the chicken embryo at concentrations of 2.5 and 5% had a favorable effect on the carcass yield. Despite showing significant differences between the groups, it should be emphasized that in the other injected groups, the value of this ratio was also at a high level and was around 75%, which means that it was higher compared to the results reported in the literature (Biesiada-Drzazga et al., 2011; Biegniewska et al., 2016; Tejeda et al., 2021). The results obtained are difficult to compare with those of other authors as there are no publications on the effect of BP administered in ovo on traits determining slaughter yield and the proportion of individual carcass elements. The works published so far have focused on prebiotics (Tavaniello et al., 2019), vitamins and amino acids (Gholami et al., 2015; El-Kholy et al., 2019), as well as glycerol and insulin (Neves et al., 2020). However, the authors of the above works found no effect of in ovo nutrition on the dissection characteristics of the bird carcass.

## CONCLUSIONS

Based on the results, it was concluded that high quality day-old chicks could be obtained by in ovo injection. A beneficial effect of 1.5% BP extract on the BW and length of day-old chicks was found. Furthermore, supplementation of chicks with 1.5% BP extract had a beneficial effect on BW during the first rearing period and showed a hypocholesterolemic effect in young chicks. At the same time, an adverse effect of a high dose of BP (5%) and a carbohydrate solution administered in ovo on the hatchability level of chicks was shown. The implementation of the carbohydrate mixture resulted in a deterioration of biochemical indices in the plasma of newly hatched chicks, especially in the case of transaminase activity.

## DISCLOSURES

The authors declare no conflicts of interest.
